# Deliberative Mapping of options for tackling climate change: Citizens and specialists ‘open up’ appraisal of geoengineering

**DOI:** 10.1177/0963662514548628

**Published:** 2014-09-15

**Authors:** Rob Bellamy, Jason Chilvers, Naomi E. Vaughan

**Affiliations:** University of Oxford, UK; University of East Anglia, UK

**Keywords:** climate change, framing risk, governance of science and technology, public participation, risk perception, technology assessment

## Abstract

Appraisals of deliberate, large-scale interventions in the earth’s climate system, known collectively as ‘geoengineering’, have largely taken the form of narrowly framed and exclusive expert analyses that prematurely ‘close down’ upon particular proposals. Here, we present the findings from the first ‘upstream’ appraisal of geoengineering to deliberately ‘open up’ to a broader diversity of framings, knowledges and future pathways. We report on the citizen strand of an innovative analytic–deliberative participatory appraisal process called Deliberative Mapping. A select but diverse group of sociodemographically representative citizens from Norfolk (United Kingdom) were engaged in a deliberative multi-criteria appraisal of geoengineering proposals relative to other options for tackling climate change, in parallel to symmetrical appraisals by diverse experts and stakeholders. Despite seeking to map divergent perspectives, a remarkably consistent view of option performance emerged across both the citizens’ and the specialists’ deliberations, where geoengineering proposals were outperformed by mitigation alternatives.

## 1. Introduction

Appraisals of deliberate, large-scale interventions in the earth’s climate system, known collectively as ‘geoengineering’, are being conducted in earnest. The idea of climate control is not new (Fleming, 2010) but has recently gained prominence in the context of anthropogenic climate change. ‘Geoengineering’ comprises an array of proposals that can broadly be divided into two types: carbon geoengineering proposals, which seek to remove and sequester carbon dioxide from the atmosphere, and solar geoengineering proposals, which seek to reflect a proportion of sunlight away from the earth (The Royal Society, 2009). These types and each proposal within them pose unique opportunities but also risk adverse impacts and unintended consequences. This has led to increasing public concern and calls for more anticipatory, reflexive, inclusive and responsive governance of geoengineering that explores possible effects and implications, and properly accounts for them in processes of technological development and decision making (Macnaghten and Chilvers, 2013; Owen et al., 2013; Stilgoe et al., 2013). Controversies over recently proposed or enacted geoengineering field experiments, such as the test-bed for the Stratospheric Particle Injection for Climate Engineering (SPICE) project (Macnaghten and Owen, 2011) and the ‘rogue’ iron fertilisation by the Haida Salmon Restoration Corporation (Tollefson, 2012), have further intensified these imperatives for public debate.

Despite these imperatives, most appraisals of geoengineering have inadequately responded to the ‘post-normal’ (Funtowicz and Ravetz, 1993) scientific context in which geoengineering resides, by employing exclusive ‘expert–analytic’ methods such as computer modelling, cost–benefit analysis, multi-criteria analysis and expert review. Through narrowly framed problem definitions, such as ‘insufficient mitigation’ efforts (reducing greenhouse gas emissions) or the risk of a ‘climate emergency’, these methods have invariably placed geoengineering in ‘contextual isolation’, marginalising legitimate mitigation and adaptation (reducing climate change impacts) alternatives (Bellamy et al., 2012). Ultimately, through a lack of reflexivity, these and other narrow framings (Bellamy, 2013) have led to geoengineering appraisals beginning to ‘close down’ on particular geoengineering proposals for responding to climate change, principally stratospheric aerosol injection: a controversial solar geoengineering idea to inject particles into the atmosphere and reflect sunlight away from the earth.

It is by now well established that highly complex and uncertain issues such as geoengineering demand that appraisals go beyond the narrow and closed forms of assessment observed to account for broader social, political and ethical issues through inclusive participation (Funtowicz and Ravetz, 1993; Stirling, 2008; Wynne, 1992). These dimensions are addressed, in part, by emerging research on public understandings of geoengineering. Media discourse analyses have begun to show how frames that might impact public perceptions of geoengineering can be more diverse than those considered in technical appraisals (e.g. Scholte et al., 2013). Other research has sought to directly elicit public perceptions of geoengineering through surveys (e.g. Mercer et al., 2011), interviews (e.g. Spence et al., 2010) and communication experiments (e.g. Kahan et al., 2012). Yet, in common with expert–analytic modes of appraisal outlined above, these studies have adopted narrow framings that close down around certain perspectives and issues.

Partly in response to these deficiencies, a literature is emerging around the use of deliberative participatory methods in geoengineering appraisal. Taking the form of focus groups or workshops, these methods have been broader in their framings and less constrained in their elicitation of participant reasonings than other appraisals. However, while adopting broader problem framings than other participatory and analytic appraisals, including situating geoengineering proposals alongside mitigation and adaptation as societal responses to climate change, deliberative engagements with geoengineering have so far not treated alternative options symmetrically, instead focusing on appraising individual geoengineering proposals (Pidgeon et al., 2013; Macnaghten and Szerszynski, 2013) or comparing those proposals against one another (Corner et al., 2013; Natural Environment Research Council (NERC), 2010). As with technically based assessments, participatory appraisals have so far been framed relatively narrowly in terms of geoengineering per se, which risks artificially isolating these proposals from wider issues of climate change. Moreover, these methods do not reconcile the need for analytic–deliberative integration (Stern and Fineberg, 1996).

This article presents findings from the first ‘upstream’ participatory appraisal of geoengineering to deliberately ‘open up’ consideration of carbon and solar geoengineering proposals alongside a range of other options for responding to climate change. We employ a novel analytic–deliberative participatory appraisal method called Deliberative Mapping (DM) (Burgess et al., 2007), which has been successfully developed and applied to analogous emerging technologies such as xenotransplantation (Davies et al., 2003) and energy-related technologies (Burgess et al., 2004). It brings together the strengths of the expert–analytic approach Multi-Criteria Mapping (MCM) (Stirling and Mayer, 2001) with those of the participatory–deliberative Stakeholder Decision Analysis (SDA) (Burgess et al., 1988). Compared to the aforementioned survey, focus group and deliberative studies on geoengineering perceptions, DM methodology has allowed us to open up the issue for the first time to a diversity of options; involve citizens, experts and stakeholders together in a symmetrical, interactive and transparent process; and engage all participants in undertaking directly comparable multi-criteria appraisals that visually map out difference and similarity of responses rather than promote aggregated outputs.

We focus, in particular, on analysing the findings of the citizens’ engagement in the DM process. The findings of the specialists’ engagement are detailed elsewhere (Bellamy et al., 2013) and drawn on, where appropriate, to further interpret and compare the citizen-based analysis. We begin by detailing the DM methods used, before presenting and discussing the results in the context of the wider literature. We conclude by reflecting on the performance of the process and outlining key recommendations for future research and policy.

## 2. Methods

**Figure 1.**
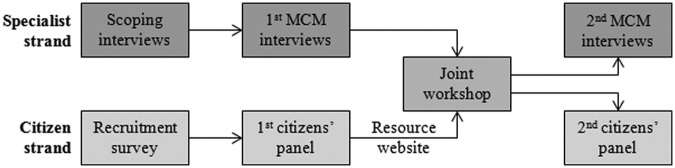
Overview of the Deliberative Mapping process. MCM: Multi-Criteria Mapping.

Taking place during the summer and autumn of 2012, the DM process comprised two parallel strands of engagement: one for citizens and the other for experts and stakeholders (specialists), with the strands converging in a joint workshop mid-way through the process (Figure 1). Following an online recruitment survey, the citizens’ strand began with a full-day citizens’ panel workshop before reconvening several weeks later for a half-day joint workshop with present specialists and a second half-day citizens’ panel workshop. Following a series of scoping telephone interviews, 12 senior experts and stakeholders were recruited who held an appreciation of the international context of climate change and a diversity of perspectives in relation to their (1) working sector (academia, civil society, industry or government), (2) disciplinary specialisms (natural or social science perspectives relating to general or specific options) and (3) personal attitudes to geoengineering research. The specialists’ strand began with 1- to 3-hour face-to-face MCM interviews before a second set of 1- to 2-hour MCM interviews several weeks later following the joint workshop with citizens (for full details see Bellamy et al. (2013)). Both strands followed a four-step multi-criteria option appraisal process in which participants (1) selected and defined options to appraise, (2) characterised a set of criteria against which they would appraise those options, (3) scored the performance of the options against those criteria and (4) assigned weightings to the criteria to indicate their relative importance.

The DM process was framed as an exercise in ‘responding to [global] climate change’ to move beyond the narrower frames of previous appraisals and broaden the context to include alternative options to geoengineering. A review produced an extensive range of options for responding to climate change, which were subsequently screened for diversity in terms of strategy, likely governance, policy instruments and novelty/maturity. As a result of this analysis, a set of seven ‘core’ options to be appraised by all participants were defined as follows:

- *Voluntary low carbon living*. Promoting voluntary reductions in domestic and commercial energy use.
- *Offshore wind energy*. Increasing the proportion of energy provided by offshore wind turbines.
- *New market mechanism*. Developing a new and expanded market-based carbon trading mechanism.
- *Biochar*. Focusing research and development on the production of pyrolysed biomass (biochar) and its application to soils.
- *Air capture and storage*. Focusing research and development on the use of technology for capturing CO_2_ from the ambient air.
- *Stratospheric aerosol injection*. Focusing research and development on the injection of reflective sulphate particles into the stratosphere.
- *Business as usual*. Continuing as usual, with no further adoption of options for responding to climate change.

The review also produced seven ‘discretionary’ options that participants were able to select and appraise at their own discretion, namely, nuclear fission energy, coal energy with carbon capture and storage (CCS), carbon tax, nuclear fusion energy, iron fertilisation, cloud albedo enhancement and space reflectors. The DM approach also allowed participants to develop and appraise their own ‘additional’ options beyond these pre-defined core and discretionary options.

In all, 14 citizens from the Norfolk (United Kingdom) public were recruited from respondents to a ‘topic blind’ online recruitment survey about ‘global environmental challenges’, after which 1 participant was unable to attend, leaving 13. While the respondents were inevitably self-selecting, a range of selection criteria were employed to ensure a diversity of perspectives among those selected for full participation. Disseminated through Norfolk’s ‘Your Voice’ scheme, the survey accrued information on each respondent’s age group, National Statistics Socio-Economic Classification (NS-SEC) and gender to ensure broad sociodemographic representation for the county of Norfolk. It elicited respondents’ perceived global issue of most concern, favoured strategy for tackling global environmental issues, given recognised sociocultural preferences (Bellamy and Hulme, 2011), and perceived cause of climate change (Table 1). Respondents with environmental ‘expertise’ were excluded from citizen recruitment, owing to such expertise gaining representation through the stakeholders in the specialist strand of the process. Each participant received an honorarium for his or her participation in the citizens’ panels and the joint workshop.

**Table 1.** Citizen participants.

Code	Age, years	M/F	NS-SEC	Issue of concern	Strategy^a^	Climate attribution^b^
P1	18–24	F	1–3	Economic downturn	H	●○
P2	25–44	M	1–3	Economic downturn	I	○
P3	25–44	F	1–3	World population	E	●
P4	25–44	M	4–9	World population	E	●○
P5	45–64	M	1–3	Climate change	I	●
P6	45–64	F	1–3	Climate change	E	●○
P7	45–64	M	4–9	World population	E	●
P8^c^	45–64	F	4–9	Climate change	E	●○
P9	65+	M	1–3	Armed conflicts	E	●
P10	65+	F	1–3	World population	H	●
P11	65+	M	4–9	Climate change	E	●
P12	65+	M	4–9	Climate change	E	●○
P13	65+	F	4–9	World population	E	●○

a Strategy refers to preferred strategy for tackling global environmental issues, where H indicates hierarchy (expert-led strategy), I indicates individualism (market-led strategy) and E indicates egalitarianism (collective-led strategy).

b Climate attribution refers to participants’ perceived causes of climate change, where ● indicates mainly anthropogenic, ○ indicates mainly natural and indicates ●○ indicates partly anthropogenic and partly natural.

c Participant only attended the first citizens’ panel due to illness during the second.

Citizen participants began their engagement with the first citizens’ panel: a 1-day workshop facilitated by the first two authors of this article. Throughout both panels, the citizens were divided into two groups by gender, for reasons of established theoretical and observational evidence of differing risk perceptions and assessments (Gustafson, 1998) and prior experience of differing engagement with the DM process (Davies et al., 2003). The first citizens’ panel was divided into several sessions: (1) openly framed group discussions of global environmental issues and climate change; (2) overview presentation of climate change, its impacts and possible responses and introduction to the core and discretionary options under consideration, followed by plenary discussion; (3) group discussions exploring options followed by poster viewing of options under consideration; (4) individual, paired and then group development of criteria for appraisal through negotiated amalgamation, followed by plenary discussion to agree upon a common set of criteria across both groups (later collated by the researcher and offered pending participant approval) and (5) preparation for the joint workshop with specialists. A purpose-built resource and debate website and printed option booklets were made available to facilitate further participant engagement in-between workshops.

The joint workshop occurred several weeks later and took place over the course of half a day. In this component, citizens reviewed ‘homework’ into the options before partaking in and then reviewing a ‘specialist workshop’. In this interactive session, they questioned the two specialists present (a volcanologist and an international conservation charity manager; the perspectives of the remaining specialists who were unable to attend were broadly introduced by the primary researcher) in order to inform their forthcoming appraisals prior to reflecting on their learning. The second citizens’ panel took place over the remaining half-day immediately after the joint workshop. In this final component, the citizens scored the relative performances of the options under consideration and assigned weightings to their criteria before reflecting on their participation in the overall DM process. The DM process produced a wealth of quantitative and qualitative data from each strand that were analysed in accordance with the procedures outlined in Burgess et al. (2007).

## 3. Results and analysis

In this section, we examine (1) how the process was contextualised and framed by citizens; (2) the existing knowledges and value frameworks drawn on by citizens in exploring the options; (3) the criteria developed by citizens and specialists to appraise the options; (4) the performance of the core options against those criteria as given by citizens, broadly contrasted with specialists scores and (5) the mapping of option performance according to citizen and specialist rankings.

### Issue framing

The men’s and women’s groups engaged with initial discussion of global environmental issues with contingent differences, with the former contextualising them within the broader issue of human overpopulation as a driver and the latter within that of human resilience to change. The particular issue of climate change emerged unprompted from both groups’ discussions early on before formal introduction by the facilitators. Both groups engaged in a discussion of climate change through its science and uncertainty as a point of entry. However, the uncertainty aspect was approached differently, with the men’s group engaging through a lens of scepticism and counter-scepticism and the women’s group engaging through a lens of trust. These lenses are consistent with recent public concerns over trust in climate science (Jasanoff, 2010), with scepticism of climate change more generally (Poortinga et al., 2011) and with gender biases (Whitmarsh, 2011). Indeed, explanations of greater scepticism among men and greater trust among women in environmental perceptions have been proffered in the latter’s greater emotional engagement (Kollmuss and Agyeman, 2002) and ability to establish connections with values and power relations (Gustafson, 1998).

Talk of scepticism and counter-scepticism in the men’s group centred around two citizens (P2, P5) who attributed climate change to mainly natural and anthropogenic causes, respectively. With reference to scientific observation and the so-called ‘Climategate’ affair, the extent and reliability of evidence were key points of contestation: ‘I still don’t believe the scientists understand a lot’ (P2) … ‘But it’s based on evidence, hard evidence’ (P5). In the absence of a ‘strong scientific background’ (P8), public trust in the arbiters of scientific knowledge was the focus of talk in the women’s group. Politicians and the media were viewed as often overstating the risks of climate change or framing their responses in ways that served particular vested interests. The women’s group also conversed about their personal experiences of weather and climate, echoing public engagements recorded in other research (e.g. Lorenzoni and Pidgeon, 2006), with older participants recollecting observed changes within their own lifetimes.

The current economic and political systems were seen as being at the core of the climate change problem by both groups. ‘Growth at all costs’ (P7), ‘materialistic’ (P1) society and a failure ‘to think long term’ (P2) were all viewed as primary drivers of climate change and global environmental issues more widely. The first session concluded with a discussion of possible options for tackling climate change, with both groups identifying sources of renewable energy and individual actions, of which the latter constituted a moral ‘responsibility’ (P3, 13) for the women’s group. Responsibility also played a larger role in relation to government and industry in both groups, in terms of the former’s power to compel change and the latter’s capacity to make change. Geoengineering was not explicitly mentioned by either group, but carbon geoengineering proposals were inadvertently referred to through large-scale afforestation in the women’s group and ‘natural processes on the Earth for sinking carbon dioxide … [such as] the sea’ (P12) in the men’s group.

### Option exploration

The two groups were invited to explore their initial reactions to the core options under consideration, in order to explore and develop options as well as elicit the existing knowledge and value frameworks they would later draw on in appraising the options. Besides the core and discretionary options, citizens noted several ‘additional’ options, all of which constituted mitigation. With the exception of personal home electricity generation, these comprised mostly forms of renewable energy, including solar energy, hydroelectric energy, wave and tidal energy and thorium energy. Large-scale afforestation, sometimes considered a carbon geoengineering option (e.g. The Royal Society, 2009), was also identified and later selected by participants for inclusion within their appraisal alongside the nuclear fusion energy discretionary option.

Citizen engagement with the geoengineering options yielded completely different reactions to those found with the mitigation options. One theme was present across all of the geoengineering options: naturalness. Public understandings of the relationship between society and ‘nature’ have long come to bear upon human developments and their impacts on the world (Franklin, 2002). Indeed, concerns over the extent to which society ‘messes’ with nature have been documented with respect to public perceptions of other emerging technologies such as genetically modified (GM) food and nanotechnology (Davies and Macnaghten, 2010; Shaw, 2002). The judgement of how natural or unnatural an option was resonates with other recent public engagement work with geoengineering, which has found naturalness to be an important determinant of perception (Corner et al., 2013). As with the aforementioned study, naturalness was a theme that was introduced unprompted by the citizens themselves; the research team avoided its introduction, given its documented strong framing effects (Corner et al., 2011).

In agreement with the study by Corner et al. (2013), the naturalness of geoengineering options was perceived differently by participants and in different contexts, contrasting with other research in which a naturalness framing was asserted. Stratospheric aerosol injection was a complex case, particularly in the women’s group, where it was simultaneously viewed as unnatural in terms of its ‘artificial interference’ (P10) but also as ‘natural’ (P13) in terms of its volcanic analogy. While biochar has been viewed as a natural process in previous research, here it was perceived as unnatural for its artificial manufacture through pyrolysis (cf. NERC, 2010). Similarly, while air capture and storage has previously been viewed as artificial and engineered, here it was perceived as natural for ‘putting [carbon dioxide] back in the ground’ (P4). These findings suggest that the naturalness of geoengineering options can be perceived and framed quite differently, depending upon the aspect or phase of operation under scrutiny.

Another cross-cutting theme was present across both of the carbon geoengineering options: scale. Both the physical scale and scalability were issues, with biochar viewed as too small scale and air capture and storage as too large scale, and both viewed as difficult to scale-up. While it was recognised that a scale of some significance would be required for these options to have an effect, foreseen difficulties at larger scales resonate with existing public concerns over large-scale renewable energy projects (Devine-Wright, 2005). This drew on other themes also raised during the discussion, primarily concerning land use in the case of biochar and storage availability in the case of air capture and storage. For biochar, these other themes concerned the option’s potential for conflict with other land uses; citizens cited existing issues with biofuels as an analogy and questioned whether such land could be used in better ways, such as through afforestation. Citizens also discussed the option’s sustainability and possible co-benefits for agriculture. For air capture and storage, other themes not only explored the safety of the option’s storage component and its potentially displeasing aesthetics but also its complementarity with other options, such as low carbon energy sources for its energy supply and the carbon market for investment return.

Other themes explored under stratospheric aerosol injection began with the issue of logic. As one citizen put it, ‘We got into this problem by pumping lots of stuff into the atmosphere, so our solution is to pump more stuff into the atmosphere?’ (P2), which resonated with remarks made by a participant in the specialist strand. Another citizen added that ‘It’s curing the symptom and not the problem’ (P5). Others remarked, ‘it’s a little bit mad scientist’ (P4) and ‘We’re getting into the realms of fantasy’ (P9). Another important theme of exploration related to the option’s possible impacts on humans and the environment, with concerns raised as to reversibility and the potential for accidents. Echoing the findings of NERC (2010), citizens raised concerns about the risk of moral hazard, whereby the use of such an option might hamper efforts in mitigation.

In contrast with the geoengineering options, citizen exploration of the mitigation options did not yield cross-cutting themes. For voluntary low carbon living, one theme was most prevalent: uptake. While it was seen as an option that both concerned and was accessible to everyone, it was also viewed as going ‘against consumerism’ (P5) and therefore unlikely to be adopted by many. Citizen participants noted education and regulation as possible ways of overcoming this inaction, with reference to industry as a priority target. For offshore wind energy, its perceived high cost and intermittent reliability were themes alongside mixed views about its environmental impacts. A low carbon footprint beyond its initial construction was contrasted with longer term environmental impacts associated with its legacy. For a new market mechanism, two themes were dominant: feasibility and enforcement. Citizens were doubtful that such a mechanism could be agreed upon, let alone operate, and concerns were expressed about corruption and fairness.

Citizen exploration of business as usual focused around three main viewpoints: that the risks of climate change brought about by this option would be serious, but humanity could adapt to some degree; that it was not an option; and that fossil fuels would be exhausted in time, irrespective of climate change.

### Criteria development

**Figure 2.**
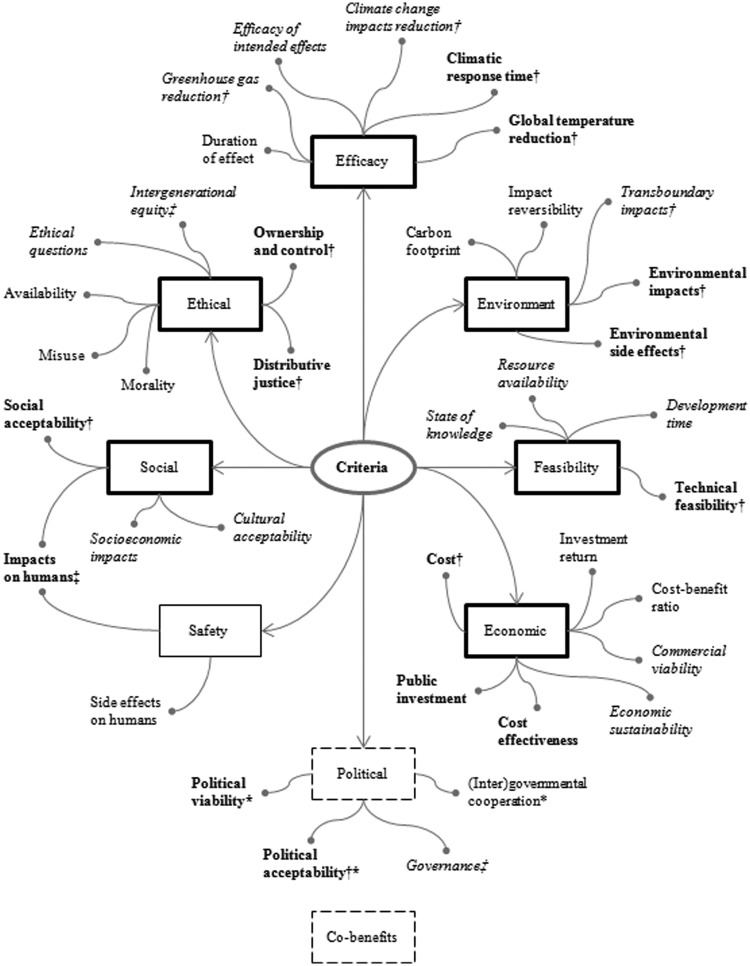
Criteria map of specialist and citizen criteria groups and subgroups. Criteria groups and subgroups in bold indicate those independently developed by both the specialists and the citizens, criteria groups and subgroups in normal font indicate those developed by citizens and dashed criteria groups and criteria subgroups in italics indicate those developed by specialists. *Criteria subgroup also developed by citizens under feasibility criteria group. Co-benefits developed as a lone but cross-cutting criterion by one specialist. Criteria groups and subgroups are high-level groupings that do not show the full complexity of issues that lie beneath. †Criteria subgroup also deployed as a principle by specialists. ‡A principle developed by specialists.

The citizens’ strand yielded a rich diversity of 32 appraisal criteria, which have been coded into 24 emergent subgroups that constitute part of seven main criteria groups (Figure 2). These groups spanned both technical and social issues, ranging from questions of efficacy, environment and feasibility to those of economics, safety, society and ethics. Specialist participants developed a total of 61 criteria, which have been coded into 29 emergent subgroups that constitute part of eight main criteria groups. These groups mirror those of the citizens’, with the exception of an absent ‘safety’ criteria group and additional ‘political’ and ‘co-benefits’ groups. While each of these issues was addressed by both strands through different criteria groups, their ‘stand-alone’ development suggests their differing relative importance to each strand. A total of 95 criteria were independently developed by the two strands, of which 80 were unique. The specialists also developed a total of 23 principles, against which options would be deemed acceptable or unacceptable and ruled in or out completely, which have been coded into 18 emergent subgroups that map onto their eight main criteria groups (Figure 2).

### Option scoring

The citizens’ appraisal of *biochar* was moderate against all of the criteria groups with the exception of safety criteria, where it scored highly. The issue of scale drove scoring against efficacy and feasibility criteria, with biochar viewed as ‘too small to make an impact’ (P6) and difficult to scale-up, respectively. The process for producing the biochar, pyrolysis, was the focus of scoring against environmental and safety criteria. Here, positive statements such as ‘I don’t see what the negative impacts could possibly be. In fact, you actually benefit in one way because you get a little bit of free fertiliser’ (P4) contrasted with comparative reference to a local, controversial waste incinerator project. While biochar was viewed socially and ethically as not likely to affect people’s lives, with the exception of its imposition on local people, its burning of biomass was seen as counter-intuitive and likely to be met with public hostility. The citizens’ moderate appraisals of biochar were mirrored by the specialists, except for where it scored highly against environment, political (expressed as a facet of feasibility by citizens) and social criteria and poorly against efficacy criteria.

*Air capture and storage* performed moderately against the citizens’ appraisal criteria groups, with the exception of the feasibility and social criteria groups, against which it scored poorly. Issues with the technology itself drove scoring against efficacy, feasibility and economic criteria, against the latter of which it was the worst scoring option overall, with issues raised concerning its perceived immaturity and high construction and maintenance requirements and costs. The perceived risk of leakages from its storage aspect and ironically high carbon footprint during construction were the focus of environmental and safety criteria. The potential for displeasing aesthetics dominated the social criteria group, while ethical criteria raised issues of fairness in relation to access to the technology, where it would be sited, who would bear the risks/benefits and where investment would originate. One participant added that in order for each of the geoengineering options to get ‘payback’ on the investments, they should be coupled with a carbon market mechanism (P2). The citizens’ moderate appraisals of air capture and storage were mirrored by the specialists, except for where it scored highly against environmental and political criteria and poorly against ethical criteria.

*Stratospheric aerosol injection* performed very poorly against citizens’ environmental criteria; poorly against safety, ethical and social criteria; moderately against feasibility and economic criteria and highly against efficacy criteria. While few citizens questioned the option’s potential efficacy for reducing global temperature, concerns were raised about the need for sustaining injections to avoid going ‘back to square one’ (P12) and its untested technical feasibility. Citizen participants also viewed the option as politically unacceptable and in need of international cooperation. While it was acknowledged in the women’s group that such ‘meddling’ (P3, P13) ‘will have negative impacts on the environment no matter what happens’ (P1), environmental and safety criteria were dominated by issues of uncertainty, with particular reference to altering weather patterns: ‘We just don’t know, so I’d err on the side of caution saying it’s very likely we’re going to have problems with this particular system’ (P4). While against economic criteria it was viewed as ‘cheap’ (P2), socially it was the worst performing option overall and was seen as only likely to be acceptable when ‘things start to get worse’ (P5) or are ‘looking bleak’ (P2). On the other hand, it was argued that ‘the media would grab hold of sulphate particles and tear it to shreds’ (P2). Issues arose quickly in relation to ethical criteria, with one citizen saying, ‘It’d be fair if it went everywhere’ (P3), with another retorting, ‘what if someone didn’t want it above them?’ (P1). This issue extended to the notion of informed consent, with another citizen asking, ‘is it morally fair to impose a solution like that on people that might not know it’s happening?’ (P12). Other citizens noted the risk of unilateral actions: ‘Country A decides what’s going to happen … and country B suffers’ (P4). Citizens’ appraisals were in agreement with those of specialists against environmental, social and ethical criteria where it scored poorly or very poorly and economic criteria where it scored moderately, but contrasted against efficacy, feasibility and political criteria where it performed poorly or very poorly for specialists.

The citizens’ appraisal of the first mitigation option, *voluntary low carbon living*, performed highly against all criteria groups, with the exception of the efficacy, environment and safety criteria against which it scored very highly. The issue of uptake was the core issue under consideration for efficacy, feasibility and ethical criteria, against the foremost of which it was the highest scoring option overall, with its high scores moderated by its being viewed as potentially insufficient, unlikely and unfair. No foreseeable issues were raised against environmental and safety criteria, against both of which it was the highest scoring option. Economic criteria, where it was the highest scoring option overall, were concerned with anticipated costs in government-led social marketing campaigns and disagreements as to whether such options would save, or cost, money for individuals. The debated extent to which it would impede lifestyles and its uneven appeal between cultures were the issue foci of social criteria. Citizens’ appraisals agreed with those of specialists against all criteria, except for political criteria where it scored moderately for specialists.

*Offshore wind energy* performed highly against the citizens’ criteria groups, with the exception of economic criteria against which it scored moderately and feasibility criteria against which it scored very highly. While its intermittency of supply was questioned under efficacy criteria, the fact that it was already in operation proved favourable against feasibility criteria, against which it was the most highly scoring option overall. Against economic criteria, it was viewed as affordable now, but that long-term maintenance would drive costs up. The options’ carbon footprint and potential risks to birds and other wildlife were raised as concerns under environmental criteria, while no issues were raised under safety criteria. Under social criteria, it was the highest performing option overall and viewed as uncontroversial in that it was a familiar technology, but citizens drew a parallel with air capture and storage, citing aesthetic concerns. Its imposition on certain locations and people was cited as ethical concerns, but it was the highest performing option against this criterion overall. Citizens’ appraisals were in agreement with those of specialists against all criteria except for efficacy and ethical criteria against which it scored moderately for specialists.

A *new market mechanism* performed moderately against the citizens’ criteria groups, with the exception of efficacy and feasibility criteria against which it performed poorly and ethical criteria against which it performed very poorly. Slow-moving negotiations towards a new market mechanism and scepticism that such negotiations would yield an agreement dominated citizens’ perceptions under efficacy, economic and feasibility criteria, against the latter of which it was the worst scoring option overall. Indeed, it was viewed as essentially sustaining business as usual under the efficacy and environmental criteria. One citizen said with respect to both the new market mechanism and offshore wind energy that they ‘would stop people using as much carbon, but all the CO_2_ … is still up there. That doesn’t include anything about removing CO_2_ does it?’ (P1). Under social criteria, it was seen as an option that did not directly affect people, but indirect impacts akin to financial crises were cited as concerns. Against ethical criteria, a new market mechanism was the worst performing option overall, with citizens citing concerns over the fairness of credit allocations and risk of abuse. Citizens’ appraisals were in agreement with those of specialists against efficacy, political, social and ethical criteria, but contrasted where specialists scored the option highly against environmental and economic criteria and moderately against feasibility criteria.

*Business as usual* performed very poorly for citizen participants against efficacy, environment and safety criteria; poorly against ethical criteria and moderately against feasibility, economic and social criteria. It was the worst performing option overall against efficacy and environment criteria, with one participant noting that ‘It’s going to be very unlikely [to reduce global warming]. But the likelihood of us going to carry on with business as usual … That’s a scary possibility’ (P5). Under feasibility criteria, differing engagements resulted in the option being seen as both feasible, as it was already in operation, and infeasible in its ability to tackle climate change. It was viewed as cheap to ‘just carry on’ (P1), but citizens also highlighted the likely escalating costs associated with living with the impacts of climate change. Against safety criteria, it was the worst performing option overall, with some views differing based on citizens’ time horizons for concern, with those concerned with nearer-term safety less concerned than those concerned with longer term safety. Citizens’ views also differed with respect to the capacity for humans to adapt to climate change. For example, two participants exchanged the following remarks: ‘We’re going to be here for a long time I reckon’ (P10) … ‘I’d like to believe that but I’ve seen too many world ending films lately’ (P3). Socially and ethically, the option was viewed as acceptable to the public by its perseverance, but that its distribution of risks on the world’s poor and on future generations would be unfair and that few would advocate it as a course of action. Citizens’ appraisals were in agreement with those of specialists against efficacy, environment, social and ethical criteria, but contrasted where specialists scored the option highly against feasibility and political criteria and poorly against economic criteria.

### Mapping option performance

**Figure 3.**
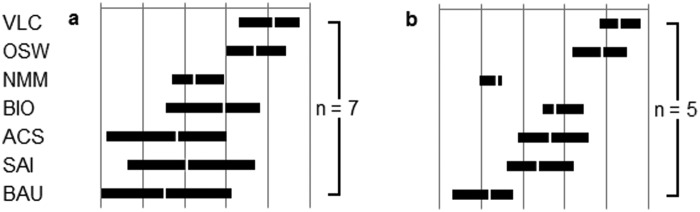
Citizens’ aggregate final rankings of core options appraised by (a) all male participants and (b) all female participants. VLC: voluntary low carbon living; OSW: offshore wind energy; NMM: new market mechanism; BIO: biochar; ACS: air capture and storage; SAI: stratospheric aerosol injection; BAU: business as usual. Frequency of participants appraising (n) is indicated to the right of the graphic. Performances increase on an arbitrary subjective scale to the right. Bar length represents the range between the most optimistic mean score of the corresponding participants and the most pessimistic mean score of the corresponding participants. The white bar dissecting the ranges is the grand mean for the corresponding participants.

The range of scores in the citizens’ appraisals allows for an overall mapping of option performance (Figure 3). Option rankings were strikingly similar across the men’s and women’s groups, with voluntary low carbon living and offshore wind energy performing much better than the other core options. While a clear pattern of consistency can be observed, so too can patterns of difference. First, a new market mechanism performs far worse in the women’s appraisal, most likely because of some misunderstanding as to what the option entailed. Indeed, obfuscations that centred around the possibility of wars over carbon credit allocations may represent a distorted ‘reconstruction’ of the concept within ‘everyday’ knowledge (Irwin and Wynne, 1996). Second, the rank order of air capture and storage and stratospheric aerosol injection is reversed, with the former viewed more favourably by the women’s group and the latter by the men’s. This reflects the qualitative data where while both groups were highly concerned by environmental, social and ethical issues surrounding the impacts and attainment of consent for stratospheric aerosol injection, the men’s group was more concerned than the women’s about the potential costs of air capture and storage. Third, a distinction between the performance, and range of scores, of the ‘technological’ geoengineering proposals and biochar can be clearly drawn in the men’s group and to a lesser extent in the women’s group, with air capture and storage and stratospheric aerosol injection performing markedly worse than biochar and with a larger range of variability. Fourth, the men’s group shows a greater range of scores than the women’s, reflecting dynamics in the latter group that sought consensus among the participants, converging scores post-deliberation.

**Figure 4.**
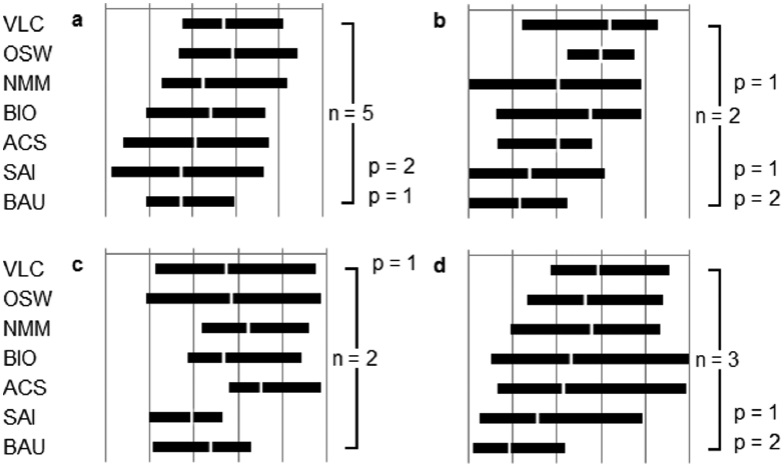
(a) Academic, (b) civil society, (c) industry and (d) government experts’ and stakeholders’ aggregate final rankings of core options appraised by all participants. VLC: voluntary low carbon living; OSW: offshore wind energy; NMM: new market mechanism; BIO: biochar; ACS: air capture and storage; SAI: stratospheric aerosol injection; BAU: business as usual. Frequency of participants appraising (n) and ruling them out on principle (p) is indicated to the right of the graphic. Performances increase on an arbitrary subjective scale to the right. Bar length represents the range between the most optimistic mean score of the corresponding participants and the most pessimistic mean score of the corresponding participants. The white bar dissecting the ranges is the grand mean for the corresponding participants.

By way of comparison, the mapping of option performance for the specialists’ appraisals is shown in Figure 4, grouped by specialist participant type (i.e. academic, civil society, industry and government) (see Bellamy et al. (2013) for further details). The key difference between these specialist actor types is that the industry sector represents a reversal of the otherwise uniform pattern. Here, the carbon geoengineering option air capture and storage is the ‘lead’ option, with mitigation options trailing behind, in contrast to most other specialist participants. Having said this, comparing Figures 3 and 4 it can be seen that the overall ranking of options reveals a remarkable degree of consistency between groups on both the citizen and specialist strands of the DM process. In the following list, we detail the ‘highest’, ‘lowest’ and ‘middle’ scoring core options across these appraisals within the process.

The consistency between strands shows two ‘highest’ performing core options, comprising mitigation options, which clearly appear to perform better than the others:

- Voluntary low carbon living;
- Offshore wind energy.

It also shows two ‘lowest’ performing options, comprising one solar geoengineering proposal, which clearly appear to perform worse than the others:

- Stratospheric aerosol injection;
- Business as usual.

The overall performance of the remaining three ‘middle’ performing options, comprising one mitigation option and two carbon geoengineering proposals, is more ambiguous:

- New market mechanism;
- Biochar;
- Air capture and storage.

## 4. Discussion and conclusion

DM of climate geoengineering against alternative options for tackling climate change has begun to open up the diverse framings, knowledges and pathways bearing upon these most complex issues to a degree that no other appraisal, analytic or participatory, has done before. First, DM’s capacity to allow citizens to frame the problem themselves has produced a broader diversity of resonant problem framings from which to conceptualise the issue, including overpopulation and human resilience to change, issues of scepticism and trust in science and deep flaws in incumbent economic and political systems. Second, its recognition of alternatives to geoengineering, spanning climate change mitigation strategies and adaptation, in addition to allowing citizens to develop their own ideas, has provided a more complete selection of options to consider. Third, by providing a more diverse and inclusive range of perspectives, it has produced a greater scope of criteria with which to scrutinise and appraise those options, which in turn has exposed different performance scores and their manifold uncertainties. As a direct consequence of this approach, a fundamentally different view of geoengineering has emerged, with geoengineering options performing lower than their mitigation counterparts. In particular, stratospheric aerosol injection has been shown to perform very poorly in stark contrast to the positive evaluations of this option found in many existing appraisals, including studies of public perceptions (e.g. Mercer et al., 2011) and especially expert–analytic assessments (e.g. Bickel and Lane, 2009; Fox and Chapman, 2011; Lenton and Vaughan, 2009).

Concurrently, a number of findings from our DM experiment are consistent with and advance those of other research into public engagement with geoengineering. The widespread uncertainties and risks involved in geoengineering, mapped here in option performance ranges and qualified by participant reasonings, are a common feature of participant discourse (e.g. NERC, 2010). Similarly, how natural geoengineering proposals appear, despite sometimes being viewed here as both natural and unnatural in different contexts, is a common lens through which perceptions are formed (e.g. Corner et al., 2013). In accordance with Macnaghten and Szerszynski (2013), we do not find the binary ‘supporters’ and ‘detractors’ of (solar) geoengineering recorded in Mercer et al. (2011), but instead find a rather more nuanced set of positions of varying levels of support or opposition under different perspectives and criteria. These positions can be understood as conditions for acceptance, expanding on those outlined in Macnaghten and Szerszynski (2013) relating to feasibility (Condition 1), side effects (Condition 2), efficacy (Condition 3) and political (Conditions 4 and 5) criteria, but they also emphasise conditions pertaining to broader environmental, economic, safety, social and ethical criteria. Expansion of conditions and criteria to include more plural normative social conditions would likely only decrease the already noted implausibility of their being met for stratospheric aerosol injection (see Szerszynski et al., 2013).

In addition to being reflexive about framing conditions, another novelty of our DM process has been its facilitation of citizen–specialist interaction through the joint workshop. This is a distinctive feature of the approach that no other existing participatory appraisal method develops in an entirely parallel and symmetrical way. The joint workshop proved to be an important step, allowing participants to substantiate and sometimes reshape many of the issues broadly identified during option exploration in the initial citizens’ panel (cf. Davies and Burgess, 2004). Rather than becoming more sceptical of geoengineering after gaining further knowledge (Spence et al., 2010), some citizens became more cautiously supportive (see also Pidgeon et al., 2013). This was not because of option or issue advocacy by the specialists present, who themselves noted their moderate positions, but because reference to actual and ongoing research was made. Such reference appears to have normalised what was previously viewed as ‘fantasy’ (P9) and got nearer to meeting conditions of feasibility and efficacy. Yet, the overall lower performance of geoengineering, particularly stratospheric aerosol injection, resonates with the low performance of other perceptively high-risk technologies appraised in the other DM processes, such as cross-species (xeno-) organ transplantation or space-based radioactive waste disposal (Burgess et al., 2004; Davies et al., 2003). Overall, the citizens engaged in a recognisably intensive learning process, but expressed regret at not having more time or information to complete the appraisal. This research has thus shown that citizens can effectively engage in complex issues such as geoengineering in the context of tackling climate change and develop informed and considered judgements that are fully comparable with those of specialists.

The DM process has opened up to broader problem definitions, alternative options and diverse perspectives and criteria and, in doing so, revealed a radically different view of option performance, posing important implications for future research and policy. However, it was limited by its resource and budgetary scale. The research was limited to 13 citizens and 12 specialists, of whom only 2 were able to attend the joint citizen–specialist workshop. While this was not intended to be statistically representative of specialists or citizens, rather to be a rich, exploratory mapping of the issues under consideration, a larger sample size and greater specialist presence at the joint workshop would have inevitably introduced a greater diversity of perspectives. Future research should therefore expand upon this social diversity, but also engage with citizens internationally for a greater cultural diversity.

It is clear from the findings of this article that no option for tackling climate change presented here or elsewhere represents a panacea, either in terms of their efficacy or in terms of any other criterion. Each option has raised unique issues, with diverse perspectives resulting in different ranges of performance and uncertainty, meaning that there is a need to discriminate between them. This is not to say that geoengineering proposals should be appraised in isolation, which would only serve to close down their appraisal, but to appraise them as discrete options alongside alternatives. Finally, our findings suggest that reflexive appraisal approaches such as DM will form a vital part of broader ambitions to build more anticipatory, inclusive, responsive and responsible ways of governing emerging technologies such as geoengineering (Macnaghten and Chilvers, 2013; Owen et al., 2013; Stilgoe et al., 2013). Recent geoengineering controversies have already highlighted the need for anticipatory appraisal, which the DM process actively attends to in an inclusive and reflexive manner. These features will help to build the responsiveness of geoengineering governance to changing natural and social conditions and expert, stakeholder and public knowledges and values, but ultimately rely upon the responsiveness of broader systems of governance and ‘meta-governance’ (Kooiman and Jentoft, 2009).
